# Enforced Higher Education

**Published:** 1898-11

**Authors:** 


### Enforced Higher Education.

The Association of American Medical Colleges, which is an organization first brought into actual existence by Dr. Eugene F. Cordell of Baltimore, has done a noble work in raising the standard of medical education in the United States, and in gaining for our country the respect and admiration of foreign medical schools and physicians. There are some schools in this country, however, which did not join this association, and which still "cut rates," so to speak, and by shortened courses and various tricks carefully omitted from mention in the annual catalogues and announcements, help to lower the standard of medical education in this country. In order to force these schools into the association and make them the equals of all the good schools, the

American Medical Association has passed very practical resolutions declining to register as delegate or permanent member any physician who shall hold the position of teacher or professor in these low-grade schools, or who shall be a graduate of such schools. If the American Medical Association has the grit to stick to this resolution, and will not be overpowered by the political members of the association, the effects of this rule will have a very salutary effect on the poorer schools.—*Maryland Med. Jour.*, July 23.

